# Pdcd2l Promotes Palmitate-Induced Pancreatic Beta-Cell Apoptosis as a FoxO1 Target Gene

**DOI:** 10.1371/journal.pone.0166692

**Published:** 2016-11-18

**Authors:** Ye Yin, Wei Yong, Jiani Yu, Xu Zhang, Haiyan Lin, Yunxia Zhu, Xiao Han

**Affiliations:** 1 Key Laboratory of Human Functional Genomics of Jiangsu Province, Nanjing Medical University, Nanjing, China; 2 Clinical Diabetes Centre of Jiangsu Province, Nanjing Medical University, Nanjing, China; 3 Department of Biochemistry and Molecular Biology, Nanjing Medical University, Nanjing, China; Broad Institute, UNITED STATES

## Abstract

Transcription factor FoxO1 is a key regulator of the insulin-signaling pathway, and is reported to play important roles in pancreatic β cell differentiation, proliferation, apoptosis and stress resistance. The multifunctional nature of FoxO1 is due to its regulation of various downstream targets. Previous studies in our lab identified potential FoxO1 target genes using the ChIP-DSL technique and one of those genes, Pdcd2l, was selected for further study. We found that the expression of Pdcd2l was increased with palmitate treatment; the luciferase assay result revealed that enhanced Pdcd2l promoter activity was responsible for the elevation of Pdcd2l expression. ChIP-PCR was performed to confirm the combination of FoxO1 to Pdcd2l promoter, result showing that FoxO1 could bind to Pdcd2l promoter and this binding was further enhanced after palmitate treatment. Overexpression of FoxO1 significantly induced Pdcd2l promoter activity, leading to increased mRNA level; consistently, interference of FoxO1 abolished the increment of Pdcd2l gene expression triggered by palmitate treatment. In addition, overexpression of Pdcd2l could further increase the percentage of apoptotic cells induced by palmitate incubation, whilst interference of Pdcd2l partially reversed the palmitate-induced apoptosis together with activated Caspase-3, indicating that the latter may play a part in this process. Therefore, in this study, we confirmed the binding of FoxO1 to the Pdcd2l gene promoter and studied the role of Pdcd2l in β cells for the first time. Our results suggested that FoxO1 may exert its activity partially through the regulation of Pdcd2l in palmitate-induced β cell apoptosis and could help to clarify the molecular mechanisms of β cell failure in type 2 diabetes.

## Introduction

Type 2 diabetes mellitus is a metabolic disease characterized by hyperglycemia, and is caused by a combination of genetic and environmental factors [1, 2]. The prevalence of diabetes has been continuously growing for the last few decades, and is becoming a worldwide epidemic [3]. Increased dietary fat intake and decreased daily physical activities are contributing to the boom in obesity, which is well known as being associated with the development of diabetes [4, 5].

Elevated plasma free fatty acid (FFA) often occurs in people with obesity, especially those with central obesity [6, 7]. Circulating FFA is known to have important physiological functions; it can be utilized by many tissues to yield large quantities of ATP. However, many studies have also suggested that an overabundance of circulating fatty acids can cause many adverse metabolic effects, most notably insulin resistance [8]. However, insulin resistance will not lead to the onset of type 2 diabetes unless it is accompanied by pancreatic β cell failure, as β-cells can respond by upregulating insulin secretion to maintain normoglycemia when insulin resistance occurs, a process known as β-cell compensation [9]. Increased nutrient supply, especially of FFA, is widely suggested as the main cause of compensatory β cell mass expansion observed in obese people [10, 11]. However, sustaining enhanced circulating FFA may aggravate insulin resistance and the chronic compensation process may promote β cell dysfunction, eventually leading to the development of type 2 diabetes [9, 12]. Chronically elevated FFA levels can also have direct negative effects on pancreatic β-cells through lipotoxicity. Long-term exposure of pancreatic islets to saturated FFA has been reported to impair not only proinsulin synthesis, but also insulin storage and secretion, and will lead to β cell apoptosis [13, 14]. Although many factors and signaling pathways have been suggested as being involved in saturated FFA-induced β cell dysfunction and apoptosis, the precise mechanisms are not fully understood.

The transcription factor FoxO1 has been reported to be a key regulator in the insulin-signaling pathway, and plays an important role in β cell function and survival. Four FoxO isoforms: FoxO1, FoxO3, FoxO4, and FoxO6 have been identified in mammalian cells; of these, FoxO1 is the most abundant, existing in the liver, adipose tissue, and β cells [15]. FoxO1 can be phosphorylated by kinases including AKT, JNKs, NF-κB, and CDK2, promoting the translocation from nucleus to cytoplasm and leading to the inactivation of FoxO1 [16, 17]. FoxO1 is a multifunction protein which has been reported to regulate metabolism, apoptosis, autophagy and cellular proliferation in various tissues [18]. FoxO1 regulates the differentiation of muscle cells and adipocytes, and also plays an important role in lipid and glucose metabolism in the liver [19–21].

A series of studies also suggested that FoxO1 has a central role in β cell differentiation, stress resistance, proliferation, and apoptosis. FoxO1 is broadly expressed in the pancreatic epithelium of the early mouse embryo, but is subsequently restricted in endocrine cells, and finally limited in β cells during pancreatic organogenesis [22]. Studies have shown that FoxO1 may negatively control β cell differentiation by regulating key transcriptional factors, including NGN-3 and NKX6-1, and siRNA knockdown of FoxO1 significantly increases insulin gene expression [23]. Ablation of FoxO1 in Nuerog3^+^ enteroendocrine progenitor cells can cause gut Ins^+^ cells to secrete insulin in response to glucose; and inducible ablation of FoxO1 in adult mice also resulted in generation of gut Ins^+^ cells, indicating that active FoxO1 is essential for preventing the differentiation of enteroendocine cells into β cells [24]. FoxO1 also plays an important role in β cell proliferation and apoptosis; it can inhibit β cell proliferation through negative regulation of PDX-1 transcription and activity. First, FoxO1 can compete with FoxA2, the positive regulator of PDX-1, for binding to the Pdx-1 promoter; second, the nuclear translocation of FoxO1 is prone to exclude PDX-1 from the nuclei [25]. FoxO1 has also been reported to prevent compensational β cell mass in insulin resistance. Constitutive nuclear expression of FoxO1 in both pancreatic ductal and endocrine β cells inhibits β cell replication without affecting insulin secretion [26]. Although FoxO1 plays a negative role in β cell replication and neogenesis, it is also required to maintain β-cell function and identity under increased metabolic stress. Mice with FoxO1 ablation in β cells exhibited hyperglycemia with a loss of β cell mass, due to β cell dedifferentiation following physical stress [27]. Overexpression of FoxO1 has been reported to act against hyperglycemia-induced oxidative stress, and prevent β cell replication in an insulin-resistant state through decreasing glucose utilization and insulin secretion, a process known as “metabolic diapause” [28]. Additionally, β-cell-specific knockout FoxO1 in *db/db* mice showed more severe glucose intolerance compared with control *db/db* mice, indicating the protective effect of FoxO1 in β cells [29]. Therefore, FoxO1 can perform different activities in response to various stimulations in β cells. The multifunctional roles of FoxO1 are probably due to its involvement in various signaling pathways. However, current studies are mainly focused on the function and regulators of FoxO1; future studies should be designed to explore the target genes regulated by FoxO1 in β cells.

Pdcd2l (programmed cell death 2-like) has the same C terminal domain as Pdcd2, which is a highly conserved eukaryotic protein with undefined function. Previous studies suggested that Pdcd2 may play an important role in cell proliferation and differentiation [30, 31]. However, little is known about Pdcd2l since there is very few study about this protein.

Previously, we used ChIP-DSL technique to search FoxO1 targets on palmitate-induced β cell apoptosis model. The results showed that the binding of FoxO1 to promoters of multiple genes changed in palmitate-induced apoptotic β cells, indicating that these genes may act as downstream targets of FoxO1 during cell apoptosis [32], and Pdcd2l was one of these target genes. In this study, we have further determined the regulation of FoxO1 on Pdcd2l expression and explored the role of Pdcd2l on palmitate-induced β cell apoptosis.

## Materials and Method

### Reagents

Fatty acid-free bovine serum albumin (BSA; fraction V), palmitic acid, Propidium iodide (PI) and Hochest-33342 were purchased from Sigma-Aldrich (St Louis, MO, USA). High-glucose Dulbecco’s modified Eagle’s medium (DMEM) and fetal bovine serum (FBS) were obtained from Invitrogen Life Technologies (Grand Island, NY, USA). The Luciferase Assay System was obtained from Promega (Madison, WI, USA). Antibodies used in this study were listed in Antibody Table (S1 Table).

### Islet isolation and cell culture

All animal studies were performed according to the Principles of Laboratory Animal Care established by the National Institutes of Health. All experiments were approved by the Research Animal Care Committee of Nanjing Medical University. Male Sprague-Dawley rats (200–250 g, purchased from the Animal Center of Nanjing Medical University, Nanjing, China) were used in the study. Islet isolation and culturing techniques have been described previously [33]. Freshly isolated islets were transferred to sterile six-well plates, and cultured in DMEM containing 11.1 mM glucose supplemented with 10% FBS. The islets were allowed to equilibrate for three h, after which they were counted and middle-sized islets with intact capsule were repacked into six well plates and cultured overnight at 37°C for further study. MIN6 cells (passages 20–30) were grown in a DMEM medium containing 15% FBS, 25 mM glucose, 50 μM 2-mercaptoethanol, 100 U/ml penicillin, and 100 μg/ml streptomycin [34]. INS-1 cells were grown in a DMEM medium containing 10% FBS, 11.1 mM glucose, 50 μM 2-mercaptoethanol, 100 U/ml penicillin, and 100 μg/ml streptomycin.

### Dissolution of palmitic acid

The 0.4 mmol/l fatty acid media was prepared as previously described, with slight modification [35, 36]. Briefly, palmitate was dissolved in ethanol at a final concentration of 0.2mol/l. The solution could be stored at –20°C for months. Before treatment of β cells, an appropriate amount of palmitate was incubated with 10% BSA at 37°C for 1–2 h, and an equal volume of ethanol was incubated with BSA as a control. The final concentration of BSA in the medium was 0.5% and the percentage of ethanol was less than 0.2%. The approximate molar ratio of fatty acids to BSA was 6:1, with 0.4 mmol/l palmitate.

### Real-time RT-PCR assay

The total RNA was extracted using a Trizol reagent. First-strand cDNA synthesis was performed using 1 μg of total RNA and an avian myeloblastosis virus reverse transcription system. The primers were designed using primer express software (Applied Biosystems, Foster City, CA, USA). Real-time quantitative PCR was performed using the SYBR Green PCR Master Mix and ABI Prism 7000 Sequence Detection System (Applied Biosystems). All data was analyzed using the expression of the gene encoding β-actin as a reference. Specific primers are listed in Table 1.

**Table 1:** The primers used in Q-PCR.

Gene name	Gene ID	Primers
β-actin (rat)	NM_031144	Forward: GAACACGGCATTGTCACCAACT
		Reverse: GCCTGGATGGCTACGTACATG
β-actin (mouse)	NM_007393	Forward:GACCTCTATGCCAACACAGTGCT
		Reverse:ACCGATCCACACAGAGTACTTGC
Pdcd2l (rat)	NM_001109544	Forward: TTGCCGCTGAGAACTGGTGT
		Reverse: GCAGGTGAAGAGCTTGGAGCTT
Pdcd2l (mouse)	NM_026549	Forward: TTCGTGGAGTGGAGAGCCTCT
		Reverse:AAGACCTAGATTAGCACTGCTGAGCAT
FoxO1 (rat)	NM_001191846	Forward: CGGAGATACCTTGGATTTTAACTTTG
		Reverse: GGTGAAGGGCATCTTTGGACT

### Western blot analysis

INS-1 cells and isolated rat islets were lysed with an ice-cold lysis buffer containing 50 mmol/l Tris-HCl, pH 7.4; 1% NP-40; 150 mmol/l NaCl; 1 mmol/l EDTA; 1 mmol/l phenylmethylsulphonyl fluoride; and a complete proteinase inhibitor mixture (one tablet per 10 ml; Roche Molecular Biochemicals, Indianapolis, IN, USA). After protein content determination using a DC protein assay kit (Bio-Rad Laboratories, Hercules, CA, USA), western blotting was performed as described previously [37].

### Apoptosis analysis

Rat islets were plated on glass coverslips within the wells of 24-well plates, and incubated with the control or 0.4 mM palmitate for 48 h in a serum-free medium. The cells were then fixed and permeabilized, and a TUNEL assay was performed according to the manufacturer’s instructions (In Situ Cell Death Detection Kit, AP, Roche) [38]. To assess the role of Pdcd2l in palmitate-induced apoptosis, plasmids or siRNA were transfected into INS-1 cells grown on glass coverslips within the wells of 24-well plates, using the NanoJuice™ transfection kit. Eighteen hours after transfection, cells were treated with 0.2 mM palmitate for another 24 h, and then stained with Hoechst 33342 and propidium iodide before being observed under an inverted fluorescence microscope [39, 40]. Cell apoptosis was determined by counting PI-positive cells against Hoechst-positive cells. Apoptotic cells were counted in three different fields under 100× vision in each well, in three independent experiments.

### Plasmid construction

A luciferase reporter construct, containing a Pdcd2l promoter, was prepared using the pGL3-promoter vector (Promega). The promoter fragments, which were about 1.5 kb in length before transcription start site of Pdcd2l, were amplified by PCR using the primers listed below (including the sites of restriction enzymes): forward, 5'-TAAGATCTGTCCCAGCATTTGGAAAGTAGT-3'; reverse, 5'- AATAAAGCTTACAACTGGGTTCTCCACG -3'. PCR products were then digested with *Bgl*II/*Hind*III, cloned into the corresponding sites of the pGL3-basic vector, and further confirmed by sequencing. Overproduction plasmids producing Pdcd2l were obtained by cloning the coding sequence (CDS) into pcDNA3.0 with *HindIII* and *EcoRⅤ*, using primers: forward, 5'- CCCAAGCTTATGGCGGCCGTCAGGAA -3'; reverse, 5'-GCGCGGGATATCCTACTTAAATAAAAATGCATCTGGGTC-3'. The FoxO1 overexpression plasmids (pCMV5-FoxO1) were obtained from our laboratory stock.

### Transient transfection and luciferase reporter assay

INS-1 cells were plated in 24-well plates 24 h before transfection. At 60–70% confluence, each well was transiently transfected with 200 ng pCMV5-FoxO1, 200 ng luciferase reporter plasmid, and 100 ng plasmid expressing the gene-encoding β-galactosidase as internal control, using a NanoJuice™ transfection kit reagent (Merck KGaA, Darmstadt, Germany) according to the manufacturer’s instructions. The cells were then incubated and harvested for luciferase reporter assays. Luciferase activity was determined as previously described [41].

### Preparation and infection of adenoviruses

The adenoviruses of the FoxO1-specific small interfering RNA Ad-siFoxO1 were obtained from our laboratory stock. The sequence and preparations have previously been described in detail [42, 43]. INS-1 cells were inoculated on 3.5 cm plates for 24 h before being infected with adenoviruses at an infection multiplicity of 200. Two hours after infection, the cells were cultured in a fresh medium for 18 h and then treated with or without 0.2 mM palmitate for another 24 h before being harvested for total RNA isolation.

### Silencing of Pdcd2l by RNA interference

The Pdcd2l-specific small interfering RNA (siPdcd2l) and control siRNA were designed and synthesized by Ribobio (Guangzhou, China). The sequence of the designed siRNA fragment was as follows: siPdcd2l, 5'- GCUCAACAGUACUAAUCUA dTdT -3'. INS-1 cells were transfected with 100 nM siRNA using a NanoJuice™ transfection kit reagent.

### ChIP-PCR assays

Four 10-cm plates containing INS-1 or Min6 cells were incubated with either control or 0.4 mM palmitate for 12 h and a ChIP assay was performed using the Chromatin Immunoprecipitation Assay kit (Upstate Biotechnology, Lake Placid, NY) according to the manufacturer’s protocol; details have been described previously [32]. The primers used are as follows: rat, forward, 5’- TTGATCCAGTCCCAATTTCAG -3’; reverse, 5’- AGTACCAGGAACACACACGGT -3’; mouse, forward, 5’- GGCTTATAATTTCCGGTCATGAT -3’; reverse, 5’- CCCAGCTCTGGCTTGAGAGT-3’.

### Statistical analysis

Comparisons were performed using students’ t tests for both groups. Data was presented as mean ± SEM. P values of less than 0.05 were considered statistically significant, and are provided in the figures.

## Results

### Treatment of palmitate increased Pdcd2l mRNA expression and protein level in pancreatic β cells

**Fig 1:**
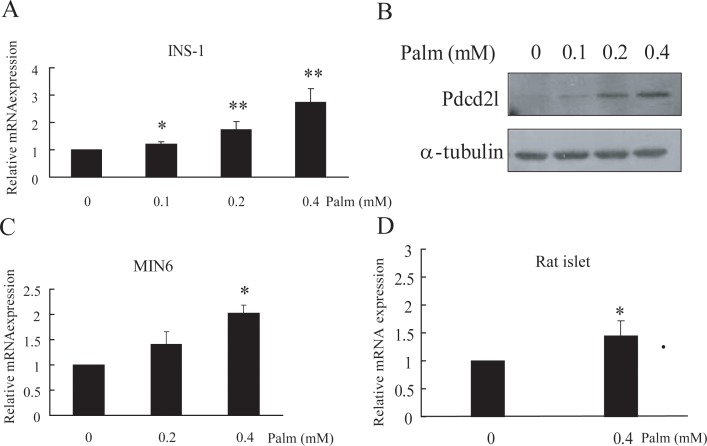
Treatment of Palmitate dose-dependently increased the mRNA level and protein level of Pdcd2l in β cells. INS-1 cells were treated with indicated dose of palmitate for 24 h, Pdcd2l mRNA level was detected by Q-PCR(A) and protein level was analyzed by western blot (B). MIN6 cells (C) and primary rat islets (D) were incubated with indicated concentrations of palmitate for 48 h, and the mRNA levels were detected by Q-PCR. Data shown are means±SEM and representative of three separate experiments. *, p<0.05 compared with control; **, p<0.01 compared with control.

INS-1 cells were incubated with different concentrations of palmitate for 48 h. As shown in the results, the mRNA expression of Pdcd2l increased dose dependently with the treatment of palmitate (Fig 1A); the protein level of Pdcd2l was also enhanced, as shown in Fig 1B. To further verify the effect of palmitate treatment on Pdcd2l expression, the mRNA level of Pdcd2l in MIN6 cells and primary rat islets was also detected; similar results were observed, as shown in Fig 1C and 1D.

### Treatment of palmitate induced β-cell apoptosis in a dose-dependent manner

**Fig 2:**
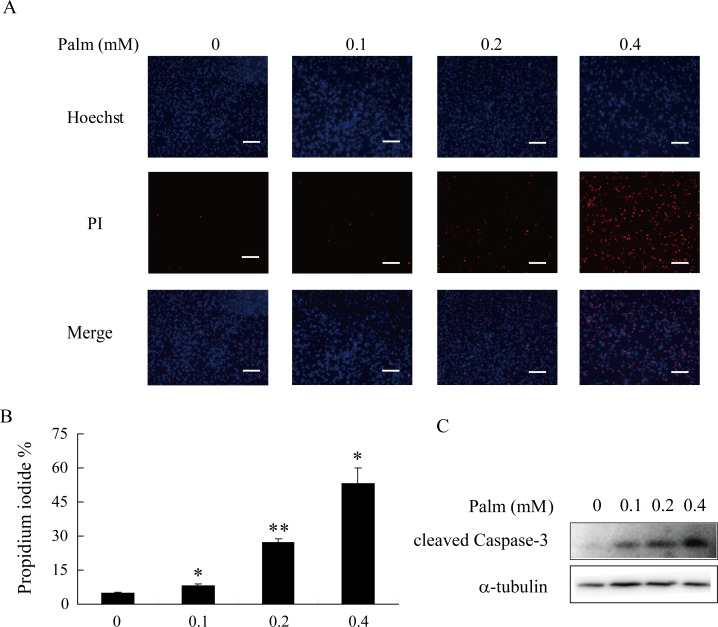
Treatment of Palmitate induced cell apoptosis dose-dependently. A: INS-1 cells were incubated with indicated concentration of palmitate for 24 h, cell apoptosis was detected by PI staining. PI-positive cells (red) indicate apoptotic cells and Hoechst-positive cells (blue) indicate live cells. Images shown here are representatives of three independent experiments. Scale bar, 20 μm. B: Quantification of the percentage of PI-positive INS-1 cells treated with palmitate. C: INS-1 cells were incubated with palmitate for 24 h, and expression of cleaved Caspase-3 was detected by western blot. Data shown are means±SEM and representative of three separate experiments. *, p<0.05 compared with control; **, p<0.01 compared with control.

Prolonged exposure to palmitate could have a deleterious impact on beta cell function and survival. Cell apoptosis was assessed with the treatment of 0.1, 0.2, or 0.4 mM palmitate for 24 h in a serum-free medium. Hoechst and PI staining results indicated that cell apoptosis was markedly increased dose dependently as shown in Fig 2A and 2B. Given that activated caspases play a crucial role in cell apoptosis, cleaved caspase-3 was also detected after palmitate treatment. As shown in Fig 2C, caspase-3 activity was also increased with palmitate incubation.

### Palmitate induced Pdcd2l mRNA expression through enhancement of its transcriptional efficiency

**Fig 3:**
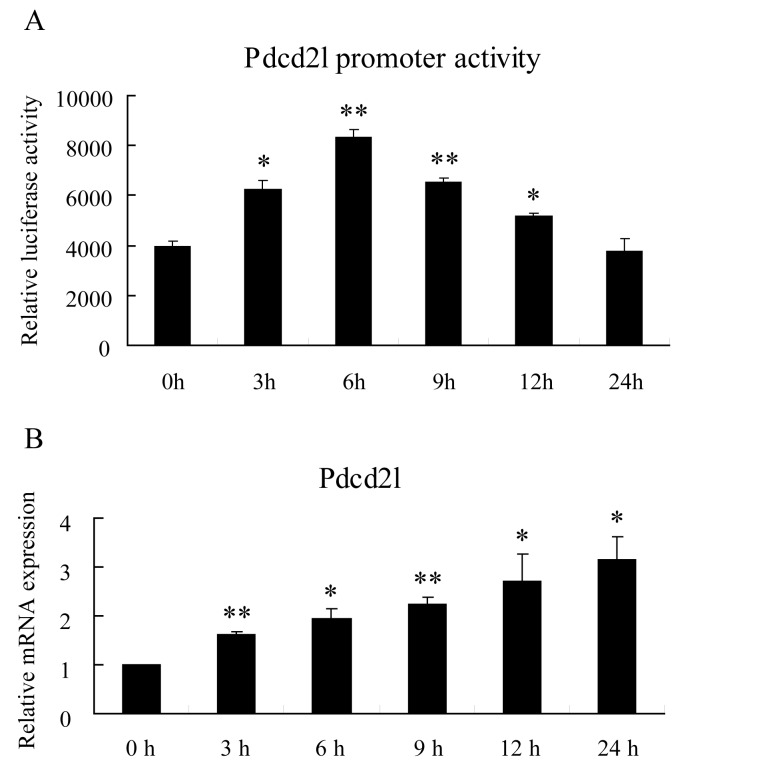
Treatment of Palmitate enhanced Pdcd2l promoter activity and gene expression time-dependently. A: INS-1 cells were transfected with pGL3-Pdcd2l luciferase reporter plasmid for 18 h and then exposure to 0.2 mol/L palmitate, cells were collected at indicated time points for luciferase reporter assays. B: INS-1 cells were incubated with 0.2 mol/L palmitae for indicated time points and gene expression was detected by Q-PCR. Data shown are means±SEM and representative of three separate experiments. *, p<0.05 compared with control; **, p<0.01 compared with control.

The above results suggest that treatment by palmitate increases Pdcd2l’s protein levels by up-regulating the gene expression. Based on this, we investigated whether palmitate induced Pdcd2l expression by increasing its transcriptional efficiency. INS-1 cells were transfected with a luciferase reporter plasmid containing a Pdcd2l promoter, and then treated with 0.2 mM palmitate. Pdcd2l’s transcriptional activity was examined by luciferase assay at various time points. As shown in Fig 3A, Pdcd2l’s promoter activity increased significantly with palmitate treatment, reaching a peak at six hours. In addition, Pdcd2l’s mRNA level showed continuous increments at the above time points, as shown in Fig 3B.

### Palmitate induced binding of FoxO1 to the Pdcd2l promoter to increase the latter’s activity

**Fig 4:**
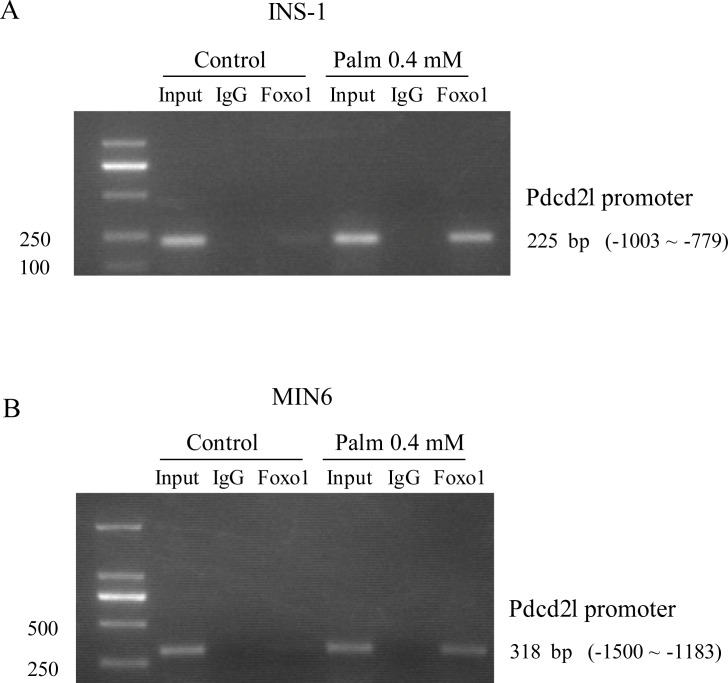
Treatment of Palmitate further increased the binding of FoxO1 to Pdcd2l promoter. INS-1 cells (A) and MIN6 cells (B) were treated with 0.4 mol/L palmitate for 12 h, and then cells were collected for ChIP-PCR. Input chromatin from control and immunoprecipitated DNA was PCR-amplified using primers specific to suspected FOXO1 target regions. Sequence enrichment in immunoprecipitated DNA from antibody (Ab, anti-FOXO1 serum) vs IgG chromatin indicated FOXO1 binding within the genomic region. Results shown here are representatives for three independent experiments.

Previous studies in our lab had reported an increased FoxO1 expression with palmitate treatment, and found that the FoxO1 increment might be involved in palmitate-induced β-cell apoptosis. In addition, in our previous research, the ChIP-DSL technique was used to identify possible target genes of FoxO1 under palmitate treatment. The results suggested that Pdcd2l is one of the target genes of FoxO1, and may play an important role during palmitate-induced β-cell apoptosis. Therefore, we first verified the specific binding of FoxO1 to the Pdcd2l promoter using ChIP-PCR. As shown in Fig 4A, FoxO1 can indeed bind to the Pdcd2l promoter and the binding was significantly increased after palmitate treatment, which indicated that FoxO1 may participate in palmitate-induced β-cell apoptosis through up-regulation of Pdcd2l expression. To rule out the possible influence of different pancreatic β cell lines, the same experiment was performed on the Min6 cell line, and similar results were obtained, as shown in Fig 4B.

### FoxO1 is involved in palmitate-induced Pdcd2l expression

**Fig 5:**
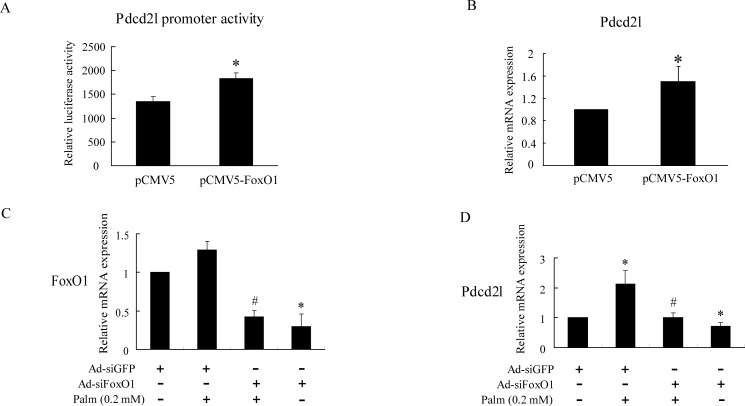
Regulation of FoxO1 on Pdcd2l promoter activity and gene expression. A: INS-1 cells were co-transfected with pGL3-Pdcd2l luciferase reporter plasmid and pCMV5 or pCMV5-FoxO1 plasmid for 24 h, and then cells were harvested for luciferase reporter assays. B: INS-1 cells were transfected with pCMV5 or pCMV5-FoxO1 for 48 h and then Pdcd2l gene expression was detected by Q-PCR. C and D: INS-1 cells were infected with Ad-siGFP or Ad-siFoxO1 for 18 h and then incubated with or without 0.2 mol/L palmitate for another 24 h, after which gene expression of FoxO1 (C) and Pdcd2l (D) were measured by Q-PCR. Data shown are means±SEM and representative of three separate experiments. *, p<0.05 compared with control; **, p<0.01 compared with control.

To further confirm the regulation of FoxO1 on Pdcd2l, we detected the effect of FoxO1 over-expression or interference on Pdcd2l expression. INS-1 cells were transfected with pCMV5-FoxO1 and pGL3-Pdcd2l; the luciferase assay result suggested that overexpression of FoxO1 significantly enhanced Pdcd2l’s promoter activity (Fig 5A). In addition, Pdcd2l’s mRNA expression was also upregulated after FoxO1 overexpression, as shown in Fig 5B. We also assessed whether interference of FoxO1 can reverse a palmitate-induced Pdcd2l increment. As shown in Fig 5C and 5D, increased Pdcd2l expression, caused by palmitate treatment, can be reversed by FoxO1 interference. The above results suggest that the participation of FoxO1 is essential for a palmitate-induced Pdcd2l increment.

### Pdcd2l participates in palmitate-induced β-cell apoptosis

**Fig 6:**
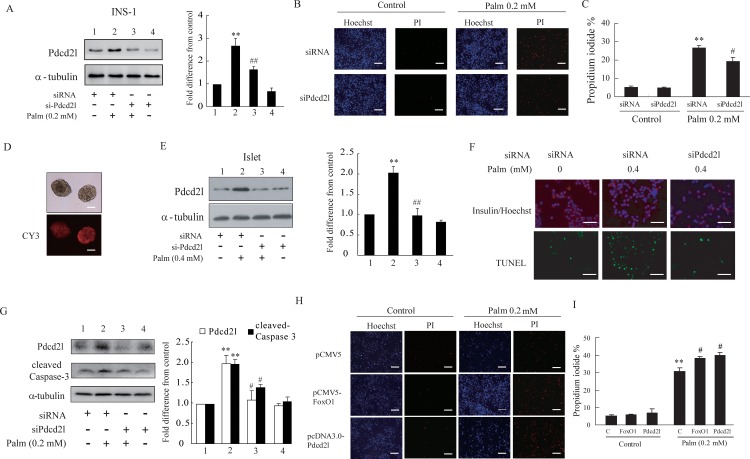
Effect of Pdcd2l in palmitate induced β cell apoptosis. A-G: Silencing of Pdcd2l partially reversed cell apoptosis caused by palmitate incubation. INS-1 cells were transiently transfected with Control-siRNA or si-Pdcd2l for 18 h, and then were incubated with or without 0.2 mol/L palmitate for another 24 h. A: Representative western blot and densitometric analysis of Pdcd2l relative to tubulin. B: Cell apoptosis was detected by PI staining. PI-positive cells (red) indicate apoptotic cells and Hoechst-positive cells (blue) indicate live cells. Scale bar, 20 μm. C: Apoptotic rate was calculated by counting PI-positive cells verse Hoechst-positive cells. G: Representative western blot and densitometric analysis of Pdcd2l and cleaved Caspase-3 relative to tubulin. D: Primary rat islets were isolated and cultured in vitro, and then transfected with Cy3-NControl (fluorescence-labeled siRNA) for 48 h to confirm the transfection efficiency. Scale bar, 100 μm. Islets were transfected with Control-siRNA or si-Pdcd2l for 24 h, and then were treated with or without 0.4 mol/L palmitate for another 48 h. E: Representative western blot and densitometric analysis of Pdcd2l relative to tubulin. F: Cell apoptosis was detected by TUNEL staining. TUNEL-positive cells (green) indicate apoptotic cells and Hoechst/Insulin double positive cells indicate live β cells. Scale bar, 20 μm. H and I: Overexpression of FoxO1 or Pdcd2l further increased cell apoptosis caused by palmitate treatment. INS-1 cells were transiently transfected with pCMV5 or pCMV5-FoxO1 or pcDNA3.0-Pdcd2l for 18 h, and then were incubated with or without 0.2 mol/L palmitate for another 24 h. H: Cell apoptosis was detected by PI staining. Scale bar, 20 μm. I: Apoptotic rate was calculated by counting PI-positive cells verse Hoechst-positive cells. Images shown here are representatives for three independent experiments. Data shown are means±SEM and representative of three separate experiments. **, p<0.01 compared with control without treatment of palmitate; ##, p<0.01 or #, p<0.05 compared with control treated with palmitate.

FoxO1 is known as a key regulator in palmitate-induced β-cell apoptosis; it may exert activity through the regulation of different target genes. Our results showed that palmitate treatment increased the binding of FoxO1 to the Pdcd2l promoter, and further increased Pdcd2l’s protein level, suggesting that Pdcd2l may play an important role in palmitate-induced cell apoptosis as a FoxO1 target gene. Therefore, we further evaluated the direct effect of Pdcd2l on palmitate-associated cell apoptosis. As shown in Fig 6H and 6I, overexpression of Pdcd2l increased palmitate-induced β-cell apoptosis; the same tendency was observed with FoxO1 overproduction. Moreover, we further determined cell apoptosis after Pdcd2l interference. As shown in the results, silencing of Pdcd2l reduced the proportion of apoptotic cells and partially protected cells against palmitate-induced β-cell apoptosis (Fig 6B and 6C). To further verify this result, primary rat islets were isolated and incubated with palmitate; the same tendency was observed in primary rat islets with Pdcd2l interference, as shown in Fig 6F. The above results reveal that increased Pdcd2l expression participates in palmitate-induced β-cell apoptosis. This finding leads us to further explore how Pdcd2l regulates cell apoptosis. Caspase-3 activation is involved in the palmitate-induced cell apoptosis progress (Fig 2C), so we further detected whether activated Caspase-3 also participates in Pdcd2l-mediated β-cell apoptosis. As shown in Fig 6G, palmitate treatment increased the cleaved Caspase-3 expression; the increment can be reversed by silencing Pdcd2l.

## Discussion

Transcription factor FoxO1 plays an important role in palmitate-induced β-cell apoptosis. Activation of FoxO1 has been identified as essential in this process, and inhibition of FoxO1 can effectively protect β-cells from palmitate-induced lipotoxicity [44]. Previous studies in our lab reported that FoxO1 activation was involved in PEG2- and Dex-induced pancreatic β-cell dysfunction, while the interference of FoxO1 significantly improved insulin secretion in β cells [37, 42, 43]. However, there are also studies suggest that FoxO1 can protect β-cells from glucotoxicity-induced oxidative stress by increasing the production of NeuroD and MafA, two important transcription factors controlling insulin2 gene expression [45]. Therefore, FoxO1 can exert opposite activities in β-cell survival and function by regulating different target genes; known about the downstream genes of FoxO1 during this process would help us to better understand the role of FoxO1 in β cell survival. However, current reports are mainly focused on the activation process and upstream regulators of FoxO1; little is known about the downstream genes regulated by FoxO1 in β cells.

Previous studies in our lab had identified FoxO1 target genes by ChIP-DSL in MIN6 cells treated with palmitate [32]. Pdcd2l, one of FoxO1 target genes according to the results, was selected for further study. We first determined the binding of FoxO1 on the Pdcd2l promoter using ChIP-PCR. The results showed that FoxO1 can bind to the promoter of Pdcd2l, and this binding was further increased after palmitate incubation (Fig 4A). Additionally, the binding of FoxO1 to the Pdcd2l promoter was further verified by EMSA, and similar results were observed (data not shown). Therefore, this result demonstrated that increased binding of FoxO1 to the Pdcd2l promoter is responsible for the enhanced Pdcd2l luciferase activity and mRNA expression that we observed (Fig 3). Moreover, interference of FoxO1 can totally reverse the increment of Pdcd2l that is induced by palmitate treatment (Fig 5). This result further supports our finding that FoxO1 upregulates Pdcd2l expression in β-cells after palmitate incubation. As shown in the result, overexpression of FoxO1 further elevated palmitate caused β-cell apoptosis, while it has little effect on untreated INS-1 cells; a similar tendency was observed with Pdcd2l overexpression. Interference of Pdcd2l can also partially reverse palmitate-induced β-cell apoptosis both in INS-1 cells and primary rat islets (Fig 6). Therefore, we conclude that FoxO1 may exert the activity in β-cell survival partially by up-regulating Pdcd2l expression.

Currently, there are very few reports about Pdcd2l, and little is known about this gene. Previous studies have reported that Pdcd2l mRNA expression was increased in Daudi cells exposed to LPS. Overexpression of Pdcd2l attenuated TNF-α releasing but upregulated IL-6 and Pdcd2 expression that was induced by LPS, suggesting that Pdcd2l may have an important role during inflammation [46]. Chen et al. reported that overexpression of Pdcd2l in HEK293 cells can inhibit cell proliferation through cell cycle arresting; transcription factors including AP-1, NF-κB, and CREB were involved in the process [47]. In our study, we found that Pdcd2l increased with palmitate treatment, and this increment was partially responsible for palmitate-induced β-cell apoptosis (Fig 1). Considering that Pdcd2l regulated cell cycle arrest in HEK293 cells, we explored whether Pdcd2l exerts its activity in palmitate induced β-cell apoptosis also through cell cycle regulation. However, neither overexpression nor interference of Pdcd2l had any effect on cell cycle arrest according to our results (data not shown).

Hyperlipidemia is also a major cause of β-cell impairment in Type 2 diabetes, in addition to hyperglycemia. Compared with unsaturated fatty acids (e.g. oleate), saturated fatty acids have been proven to be more toxic to the pancreatic islets. Palmitate, as the most abundant saturated fatty acid in human plasma, can induce cell apoptosis in various β-cell lines and primary pancreatic islets. Cellular stresses, such as ER stress and oxidative stress, can be induced by palmitate; these stresses can further promote β-cell death through intrinsic apoptosis pathways [48, 49]. Activation of caspase cascades, such as caspase-9 as well as downstream caspase-3 and caspase-7, could eventually cause cell apoptosis [50]. As shown in our results, treatment of palmitate for 24 h triggered cell apoptosis dose-dependently, accompanied by elevated cleaved caspase-3 expression (Fig 2). In addition, our findings suggested that Pdcd2l interference reversed palmitate-induced β-cell apoptosis through inhibition of caspase-3 activation (Fig 6). However, whether Pdcd2l exerts a direct or indirect effect on caspase-3 activation still needs to be explored.

In this study, we identified Pdcd2l as a FoxO1 target gene in β cells for the first time. We also found that palmitate triggered Pdcd2l expression through FoxO1, and that increments of Pdcd2l may contribute to palmitate-induced β-cell apoptosis through regulation of caspase-3 activation. Our study has discovered a new gene participating in saturated fatty acid caused β-cell death. This finding will help us to better understand the molecular mechanism of lipotoxicity-induced β-cell loss and impairment, and provides a new target for the treatment of type 2 diabetes.

## Conclusions

Our results found that palmitate treatment could increase Pdcd2l gene expression dose-dependently in β cells. FoxO1 is an upstream regulator of Pdcd2l and is required for palmitate caused Pdcd2l increment. FoxO1 may exert its activity partially through the regulation of Pdcd2l in β cells. Pdcd2l participated in palmitate induced β cell apoptosis, and interference of Pdcd2l can partially protect β cell from apoptosis. Our study had identified Pdcd2l as a novel target of FoxO1 and explored the role of Pdcd2l in β cell apoptosis for the first time.

## Supporting Information

S1 Table Antibody. (DOC)
