# Supplementary material for: Pdcd2l Promotes Palmitate-Induced Pancreatic Beta-Cell Apoptosis as a FoxO1 Target Gene
Source: PLoS One. 2016 Nov 18;11(11):e0166692. doi: 10.1371/journal.pone.0166692 (PMC5115776; doi:10.1371/journal.pone.0166692)
Supplement: S1 Table — (DOC) [file pone.0166692.s001.doc]

| Peptide/protein target | Antigen sequence (if known) | Name of Antibody | Manufacturer, catalog #, and/or name of individual providing the antibody | Species raised in; monoclonal or polyclonal | Dilution used |
| --- | --- | --- | --- | --- | --- |
| FoxO1 (Western Blot) |  | FKHR Antibody | Santa Cruz, sc-11350 | Rabbit, polyclone | 1:1000 |
| Pdcd2l |  | Pdcd2L Antibody | Santa Cruz, sc-101251 | Mouse, monoclone | 1:500 |
| α-Tubulin |  | α/β Tubulin | Cell Signaling, 2148 | Rabbit, polyclone | 1:1000 |
| Caspase 3 |  | Cleaved Caspase 3 | Cell Signaling, 9664 | Rabbit, monoclonal | 1:1000 |
| FoxO1 (chIP) |  | FKHR Antibody | Santa Cruz, sc-11350 X | Rabbit, polyclone | 1:1000 |
